# Effects of Premedication with Metoprolol on Bleeding and Induced Hypotension in Nasal Surgery

**DOI:** 10.5812/kowsar.22287523.3408

**Published:** 2012-01-01

**Authors:** Poupak Rahimzadeh, Seyed Hamid-Reza Faiz, Mahmoud Reza Alebouyeh

**Affiliations:** 1 Department of Anesthesiology and Pain Medicine, Tehran University of Medical Sciences (TUMS), Tehran, Iran

**Keywords:** Metoprolol, Hypotension, General Surgery

## Abstract

**Background:**

One of the major problems in surgery is intraoperative bleeding which reduces visibility in the operative field. An important task for an anesthetist during head and neck surgery is to improve intraoperative visibility.

**Objectives:**

The purpose of this study was to compare the amount of bleeding using different doses of oral metoprolol during three common types of nasal operation; rhinoplasty, septoplasty and functional endoscopic sinus surgery, as this is one of the complications during head and neck surgery.

**Patients and Methods:**

In a randomized, controlled, open clinical trial, 88 patients who were candidates for nasal operations were studied. Patients entering the study were divided into four groups and randomly assigned to receive 50 mg metoprolol a night before the operation, 50 mg metoprolol on the day of operation, 50 mg metoprolol on the night and on the day of operation, or a placebo. Following the patient’s preparation on the operating table and after intubation, systolic and diastolic blood pressures were measured in a non-invasive oscillometric way, and their pulse rate was recorded simultaneously. All the data were recorded during the surgery as well. Bleeding was measured by the quality scale proposed by Formme and Boezaart.

**Results:**

There was a statistical significance between using metoprolol and the amount of intraoperative bleeding. All patients who received metoprolol the night before surgery and on the day of surgery had slight bleeding during the surgery. In addition, there was a statistical significance between patients’ agitation levels and the time they received metoprolol.

**Conclusions:**

Decreases in both systolic blood pressure and heart rate to less than 60 beats per minute reduces intraoperative bleeding. These rates can be achieved by using beta-blocker drugs. In this study, using a double-dose of metoprolol significantly reduced intraoperative bleeding and improved the quality of the operative field. It also reduced patients’ agitation in the recovery room.

## 1. Background

Excessive bleeding in the surgical field during an intervention appears to be one of the most serious problems of head and neck surgery. Incomplete blocks may be associated with topical anesthesia of head and neck, which can lead to a requirement for multiple injections and discomfort for both patients and doctors. In these types of surgery general anesthesia is often preferred over topical anesthesia. In addition, general anesthesia allows hypotensive anesthesia to be achieved (1, 2). The unique and complicated anatomical structures of the head and neck, along with their proximity to the cranial base, brain, eyes, blood vessels and nerves requires the surgeon to have detailed anatomical knowledge and the ability to accurately identify structures. It is also very important for the surgeon to have a good view during surgery (1, 3).

Inappropriate bleeding is one of the complications of nasal surgeries because it can lead to poor visibility for the surgeon and visibility is further reduced the longer the time of the intervention is extended. Increased bleeding can also cause surgeries to finish before they are fully completed. Improvement of intraoperative visibility by reducing bleeding is an important task for an anesthetist during head and neck surgery (2, 4, 5). Studies show that using beta-blockers before surgery reduces long term cardiovascular complications and intraoperative bleeding (6, 7). The probable mechanism of beta-blockers in hemodynamic control is a reduction and attenuation of the excitatory effect caused by a sudden increase of catecholamine during surgery. It is believed that beta-blockers are responsible for improvement of the cardiovascular condition and patients’ hemodynamic stability via changes in stress related physiological response (5, 8). For this purpose we studied the effect of beta-blockers and the patient’s position on the surgical table to induce hypotension (9, 10). A number of different beta-blockers at different doses have already been tested, but we chose an easily obtained drug, oral metoprolol for this study. Vasodilators, beta-blockers, higher dose of inhalational or intravenous anesthetics, or a combination of all of these agents are the main substances which are used to achieve controlled induced hypotension. Induced hypotension produced by using an anesthetic agent requires it to be applied at a higher dose, which may cause postoperative complications (11).

## 2. Objectives

In this study deliberate hypotension was induced with reduction of peripheral vessel resistance and decrease in heart rate.

## 3. Patients and Methods

We conducted a randomized, controlled, open-clinical trial for 18 months at the Rasoul Akram Medical Center. 88 patients including both male and female patients’ (ASA physical status I and II), aged 16 to 45 years, weight 40 to 95 kg, undergoing rhinoplasty, septoplasty, and functional endoscopic sinus surgery were studied. Because it was an open trial, all the patients who underwent the above-mentioned operations during the 18 month period and who were able to take metoprolol were included in this study. The study did not include patients with cardiovascular system pathology, cardiac failure, chronic hypotension, as well as patients with blood system pathology, coagulation disorder, anemia (Hb < 10 g/dL) and those prescribed aspirin or other medications affecting their coagulation system. Patients with kidney or liver dysfunctions were not included. Before surgery the patients’ blood cell count, urea, serum creatinine, and prothrombine time (PT) were recorded. Because each surgeon only operated on one type of the above-mentioned surgeries, three different surgeons participated in this study. The study protocol was approved by institutional research committee and patients also gave their informed written consent.

Patients who received metoprolol entered the study, and some patients were picked as the control group. Patients entering the study were divided into four groups and randomly and blindly assigned to receive: 50 mg metoprolol at night before the operation, 50 mg metoprolol on the day of operation, 50 mg metoprolol at night and on the day of the operation, or a placebo. All patients received premedication of 10 mg oxazepam and 150 mg ranitidine on the night and on the day of operation. For all patients the bed-head of the surgical table was raised 30°. After preparation and pre-oxygenation, patients were first pre-medicated with an infusion of midazolam 0.05 mg/kg, fentanyl 1 µg/kg, lidocaine 1 mg/kg. Induction of anesthesia was achieved with an infusion of thiopental 5 mg/kg and atracurium 0.5 mg/kg and after 3 to 5 minutes patients were intubated with the appropriate tube size. Anesthesia was maintained with an infusion of propofol (100 µg/Kg/min), in the 50:50 percent mix of oxygen and nitrous oxide anaesthesia. Following induction of the anesthesia, for patients in all groups, an infusion of 10 mg of atracurium per 30 minutes and 50 µg of fentanyl per hour were continued. After the patient’s preparation on the surgical table and following intubation, their systolic and diastolic blood pressure were measured in a non-invasive oscillometric way. Frequency of heart contractions was also recorded simultaneously.

In addition to an infusion of adrenalized lidocaine for all the patients, before starting surgery, surgeon infiltrated 1-2 mL 1% lidocaine with adrenalin 1/100,000 submucosally into the lateral nasal wall. During surgery systolic and diastolic blood pressure were measured every 15 minutes and pulse rate was recorded. These average values were analyzed for statistical evaluation. Cardiac electrical activity and end-expiratory CO2 were measured as well. To evaluate the quality of the operative field during surgery, in addition to blood-gas analysis, volume of sucked and collected blood, and the surgeon’s assessment of operative field condition, the quality scale proposed by Fromm and Boezaart was also used (1):

1. Grade 0: No bleeding
2. Grade 1: Slight bleeding - no blood suctioning required.
3. Grade 2: Slight bleeding - occasional suctioning required.
4. Grade 3: Slight bleeding - frequent suctioning required. Operative field is visible for some seconds after evacuation.
5. Grade 4: Moderate bleeding - frequent suctioning required. Operative field is only visible immediately after evacuation.
6. Grade 5: Severe bleeding - constant suctioning required. Bleeding appears faster than can be removed by suction. Surgery is hardly possible, and sometimes impossible.

Surgical time was recorded from the first injection of regional anesthetic to the end of surgery. If a patient had excessive bleeding or a patient required additional medication to control blood pressure, an infusion of remifentanil was used and recorded. 10 to 15 minutes before ending surgery and after recording the surgeons’ opinion regarding the surgical field, the anesthetic agent was decreased. Patients’ agitation in the recovery room was assessed based on the Richmond Agitation Sedation Scale (RASS). Grades from 0 to 4 were assigned to the four levels of patient’s agitation: alert and calm, anxious, apprehensive, very apprehensive, and aggressive. All the patients after receiving discharge criteria from recovery were transferred to the ward.

## 4. Results

Eighty eight patients were involved in this study. If data was in a normal distribution, one-way ANOVA was used to compare groups. Ordinal variables were also assessed with one-way ANOVA using a Tukey’s post-hoc test for normally distributed data and with a Kruskal–Wallis one-way ANOVA on ranks with Dunn’s post-hoc test for data non-normally distributed, a significance level of P < 0.05 was selected. Epi Info 6 software was used to determine relative risk. Demographic findings of the study; age, gender, and type of operation (ASA physical status classification) are shown in Table 1. All data was analyzed with SPSS 13 (Statistical Package for the Social Sciences) software. The study group was comprised of four groups of patients who received metoprolol at different times. 28 patients received a single-dose of metoprolol on the day of their operation, 36 patients received a single-dose of metoprolol on the night before the operation, 16 patients received a double-dose of metoprolol on both the night and the day of operation, and 8 patients in the control group received no metoprolol.

**Table 1.** Patients’ General Characteristics

Variable	Mean ± SD	No. (%)
Age	25.9 ± 7.5	-
Gender		
Female	-	36 (40)
Male	-	
BMI ^a^	21 ± 2.1	-
Operation		
Nasal sinus functional endoscopy	-	30 (36)
Rhinoplasty	-	40 (44)
Septoplasty	-	18 (20)
ASA ^a^		
1	-	86 (96)
2	-	2 (4)
Time of receiving metoprolol		
P ^a^	-	8 (8)
M ^a^	-	28 (30)
N ^a^	-	36 (46)
MN ^a^	-	16 (16)

^a^ Abbreviations: ASA, American society of anesthesiologists physical status classification system; BMI, body mass index; M, morning before operation; MN, morning and night before operation; N, night before operations; P, placebo

The amount of bleeding, patient’s agitation, surgeon’s assessment of the operative field condition, and requirements for medication to control bleeding or in order to control blood pressure were measured upon receiving metoprolol (Table 2 and 3).

**Table 2.** Comparison of Results When Receiving Metoprolol at Different Times

Variable	M ^a^	N ^a^	MN ^a^	P ^a^	*P* value
Bleeding, No.					0.029
1 (slight)	16	4	16	1	
2 (low)	8	1	-	4	
3 (moderate)	4	16	-	3	
4 (severe)	-	6	-	3	
Agitation , No.					0.239
1 (restless)	24	20	16	6	
2 (agitation)	6	16	-	2	
Surgeon satisfaction , No.					0.071
No satisfaction	12	12	-	4	
Satisfied	16	24	16	4	
Need for additional drugs to control bleeding or blood pressure , No.	16	24	_	6	0.571

^a^ Abbreviations: M, morning before operation; MN, morning and night before operation; N, night before operation; P, placebo

**Table 3.** Relative Risk of Surgical Complications in Metoprolol Recipients and Control Group

Variable	Relative Risk	95% CI ^a^	*P* value
Severe bleeding , No.	0.56	0.37-0.86	0.05
Agitation , No.	1.14	0.33-3.98	0.412
No surgeon satisfaction , No.	0.60	0.28-1.30	0.24
Need for additional drugs to control bleeding or blood pressure , No.	0.67	0.42-1.05	0.17

^a^ Abbreviation: CI, confidence interval

The results showed that there is a statistical significance between the amount of surgical bleeding and receiving metoprolol (P = 0.029). The results are shown in Table 3. Receiving metoprolol reduces intraoperative bleeding with a relative risk of 0.56 but the differences in the time patients received metoprolol had no effect on the amount of bleeding. All patients who received the double-dose of metoprolol on both the night and day of their surgery had slight bleeding (Grade 2 on the Fromm and Boezaart scale). There was no significant decrease in the amount of bleeding for the patients who received a single-dose of metoprolol on the night before surgery or on the day of their surgery. Moreover for all the patients who received a double-dose of metoprolol there was no need to use additional medication to control blood pressure or to control bleeding (Table 2). Even though in comparison with the other groups, patients who received a double-dose of metoprolol did not need to receive additional medication, in the statistical evaluation this difference was not statistically significant. The relative risk of all the effective factors on surgery is shown for the groups that received metoprolol and the control group in Table 3. Results showed that the only effective factor in lowering the amount of bleeding is metoprolol. At the time they received metoprolol, patients’ hemodynamic changes during different stages of surgery are shown in Table 4. There is no significant statistical difference between the systolic blood pressure in the three groups of patients who received metoprolol, in different phases of the operation, which are; before anesthesia, after intubation, and one hour after starting the surgery. No drop in systolic blood pressure was observed (Table 4). Changes in the diastolic blood pressure in patients who received a double-dose of metoprolol were not statistically significant but in other groups this decrease was significant (Table 4). Before the operation patients’ heart rates were different. Patients who received a double-dose of metoprolol showed the lowest heart rate while patients who did not receive metoprolol showed the highest heart rates (Table 3). Finally the duration of the surgery for all four groups was similar and there was no significant statistical difference.

**Table 4.** Summary of Hemodynamic Parameters at the Time of Receiving Metoprolol

	M ^a^	N ^a^	MN ^a^	None^b^	*P* value
SBP ^a^, Mean ± SD					
Before intubation	112.3 ± 12.6	121.8 ± 12.5	111.2 ± 14.5	117.5 ± 12.5	0.102
Post intubation	103.6 ± 14.3	109.1 ± 12.7	120.2 ± 29.1	107.5 ± 9.5	0.170
Intra operation	95.4 ± 10.2	91.3 ± 6.5	86.2 ± 7.4	90.1 ± 8.1	0.089
DBP ^a^, Mean ± SD					
Before intubation	71 ± 7.1	74.1 ± 7.3	70.1 ± 9.2	70 ± 8.1	0.450
Post intubation	66 ± 7.3	71.3 ± 7.1	72 ± 10.8	67.5 ± 9.5	0.185
Intra operation	61.6 ± 3.6	60.4 ± 2.1	15.71 ± 2.5	60 ± 0	0.003
PR ^a^, Mean ± SD					
Before intubation	76.4 ± 13.1	86.3 ± 13.4	74 ± 11.1	94 ± 9.5	0.013
Post intubation	81.2 ± 12.7	84.6 ± 13.5	76.5 ± 10.1	93.7 ± 11.1	0.145
Intra operation	72.4 ± 11.4	77.4 ± 11.5	66.5 ± 8.71	84.1 ± 9.3	0.036

^a^ Abbreviations: DBP, diastolic blood pressure; M, morning before operation; MN, morning and night before operation; N, night before operations; PR, pulse rate; SBP, systolic blood pressure

^b^ No drug usage

## 5. Discussion

Controlled induced hypotension plus controlled stress related hemodynamic response during head and neck surgery decreases intraoperative bleeding and improves visibility of the operative field (1, 2, 4, 7). Koshing described the first use of induced hypotension as a method for decreasing intraoperative bleeding in 1917. In 1946, Gardner applied this method for arteriotomy and for a number of decades this method found an increasing role in anesthesiology (12). Induced hypotension achieved by using only inhalation or intravenous anesthetic agents requires the application of higher doses, which causes recovery time to be prolonged. So in order to achieve the desired level of hypotension in addition to an anesthetic agent, additional hypotensive drugs are the preferred method (11). Recent studies show that a decrease in systolic blood pressure and heart rate to less than 60 beats per minute reduces intraoperative blood loss (4, 12). Based on the investigation results, beta-blocker drugs have these two characteristics and with proper dosage levels have been used to induce hypotension. According to this study’s findings, using a double-dose of metoprolol reduces intraoperative bleeding significantly and improves the visibility of the operation field which increases surgeons’ level of satisfaction. Moreover it reduces patients’ level of distress in the recovery room. However using a single-dose of metoprolol in the control group had no effect on reducing intraoperative bleeding (13).

Evidently, in order to control the cardiovascular hemodynamic response and manage catecholamine levels related to surgical stress, using repetitive or a higher dose of metoprolol is required. In other words, in order to improve the condition of the candidate for head and neck surgery operations with less bleeding and less stress on both surgeon and anesthesiologist, using metoprolol leads to a positive homodynamic response and achieves the desired blood pressure. It needs to be administered several days before the surgery or even on a pre-operation visit and is also required on the day before surgery (4, 5).

According to Boezaart and his colleagues, an ideal category scale value for surgical conditions is between 1 and 2 (1). In this study, using a double-dose of metoprolol a grade 2 of bleeding on the value scale was achieved. Because of its cardio-selective effects and its particular effect on class one beta-receptors, which inhibits its non-selective effect on other blockers, and as it has no serious effects such as; reducing blood pressure, sedation, and severe bradycardia, it is suggested that drugs from the metoprolol family are used to reduce intraoperative bleeding (14).

The results show that using the appropriate dose of metoprolol improves the operating field condition and reduces undesirable hemodynamic responses. Although further studies are required to gather further information about the precise dose and times for using these metoprolol family drugs. The authors of this article are attempting to carry out more studies on this drug family for this purpose and they encourage other colleagues to research this issue as well.
